# Appropriateness of radiological diagnostic tests in otolaryngology

**DOI:** 10.1186/s13244-022-01263-y

**Published:** 2022-08-04

**Authors:** Antonio Almodóvar, Elena Ronda, Raquel Flores, Blanca Lumbreras

**Affiliations:** 1 General University Hospital of Elche, Elche, Spain; 2 CIBERESP, Madrid, Spain; 3 Public Health Research Group, University of Alicante, Carretera San Vicente del Raspeig s/n, San Vicente del Raspeig, 03690 Alicante, Spain; 4 Department of Public Health, History of Science and Gynaecology, Miguel Hernandez University, Elche, Spain

**Keywords:** Otolaryngology, Diagnostic imaging, Investigation appropriateness, Radiation dosimetry

## Abstract

**Objective:**

To evaluate the appropriateness of imaging tests associated with radiation in the field of otolaryngology according to the available recommendations, and to estimate the effective radiation dose associated.

**Method:**

Cross-sectional epidemiological study of the totality of the imaging test requests carried out by two Spanish hospitals (*n* = 1931). We collected the following information: patient demographic data, type of imaging test, imaging tests referred in the previous 12 months, referrer department and diagnostic suspicion. In accordance with the available guidelines, we considered the requests: (a) Appropriate; (b) Inappropriate; (c) Not adequately justified; (d) Not included in the guidelines. We calculated the prevalence of each category and their variation according to the different variables. Collective and per capita effective dose were calculated for each category.

**Results:**

Of the 538 requests, 42% were considered appropriate, 34.4% inappropriate, 11.9% not adequately justified and 11.7% not included in the guidelines. Imaging tests requested by general partitioners (aOR: 0.18; 95% CI: 0.06–0.50) and clinical departments (aOR: 0.27; 95% CI: 0.11–0.60) were less likely to be considered appropriate than those requested by the Otolaryngology department. Patients with a diagnosis suspicion of tumour pathology were more likely to have a requested imaging test classified as appropriate (aOR: 7.12; 95% CI: 3.25–15.61). The cumulative effective dose was 877.8 mSv, of which 40% corresponded to tests classified as inappropriate.

**Conclusions:**

A high percentage of imaging tests are considered as inappropriate in the field of otolaryngology, with a relevant frequency of associated effective radiation dose. Type of department, the diagnostic suspicion and the type of imaging tests were variables associated to the inappropriateness of the test.

## Key points

- More than 40% of imaging tests in otolaryngology were considered as inappropriate.
- X-rays showed a much higher rate of inappropriateness than CT.
- A high percentage of imaging test requested do not include sufficient clinical information.

## Introduction

Diagnostic X-ray imaging tests and nuclear medicine represent the main source of unnatural radiation to which the general population’s is exposed [1]. Exposure can lead to different types of cancers [2, 3] and to other effects, such as immunosuppression [4] or genetic alterations [5].

Given the growing frequency of these tests and their associated risks, different strategies have been developed to reduce their use, and the resulting exposure. The Federation Drug and Administration (FDA) has published an initiative to reduce unnecessary exposure to medical radiation [6]. This initiative includes measures such as the promotion of the use of safe medical tests and the need for informed clinical decision-making. To support this decision-making, it is necessary to increase patient awareness and to create a radiation dose register. The European Commission has also promoted initiatives to control the risks of exposure to ionising radiation, the most relevant of which is the Directive 97/43/EURATOM [7]. In Spain, although a regulation was developed in 2019 to implement this directive [8], it has not been applied in clinical practice. However, several recommendations have been established in different settings to guide clinicians when they order an imaging test [9].

According to a previous 12-year study [10], carried out between 2007 and 2018, nearly 5% of patients, 2.5% of whom were in the 0–20 year age group, received doses higher than 50 mSv. In addition, one third of the tests associated with radiation carried out in clinical practice were considered inappropriate according to the available recommendations [11]. Both the general population and a relevant percentage of clinicians overlook the risks associated with exposure to medical radiation and are not aware of the established recommendations to reduce this exposure [12, 13]. Efforts carried out to reduce exposure to medical radiation have thus failed to influence clinical practice, making it necessary to take action to implement these initiatives.

In otolaryngology (ORL), imaging tests are frequent, and several associations have developed recommendations to guide their appropriate use. The American Academy of Otolaryngology [14] has published guidelines regarding imaging tests in which the benefit-risk balance is negative, dubious and/or of reduced clinical value. The Spanish Society of Otolaryngology participated in the 'Do Not' initiative with the aim of reducing the use of unnecessary health interventions [15].

However, to date, no evaluation has been performed regarding the follow-up of these recommendations in the field of ORL. Nor has there been any assessment of the percentage of performed imaging tests considered inappropriate based on the clinical practice guidelines available. The objective of this study was to evaluate the appropriateness of imaging tests associated with radiation in relation to the recommendations issued in the field of otorhinolaryngology and the associated effective radiation dose, as well as the factors associated with appropriateness.

## Methodology

Cross-sectional epidemiological study to analyse the appropriateness of imaging tests associated with ionising radiation in ORL in two public hospitals in the Valencian Community in Spain. The study followed the STROBE guidelines [16].

### Population

The target population was the residents in the catchment area of two hospitals: the Vega Baja Hospital (catchment area: 199,103 inhabitants) and the General University Hospital of Elche (catchment area: 163,434 inhabitants). These are reference hospitals for all individuals living in their catchment areas and belong to the National Health Care System, which covers 98.5% of the Spanish population.

### Sample size

Based on previous studies, 52% of imaging tests performed in clinical practice were considered inappropriate with respect to diagnostic suspicion [11]. A minimum of 384 tests must be included with an accuracy of 5% and a confidence interval of 95%.

### Selection criteria

We included all imaging test requests carried out between July and October 2019 to patients of any age and relating to the ORL pathology or anatomical area: ear and temporal area, pharynx, larynx, thyroid gland, salivary gland and neck and nostril, paranasal sinuses and adjoining facial areas requested by any department (primary or specialised) and by any clinical specialty. The year 2019 was selected to avoid including the COVID-19 pandemic period and its impact on health care and clinical practice [17]. Tests with no associated radiation (Magnetic Resonance Imaging and Ultrasound) were excluded.

During the study period, a total of 1,931 tests that met these criteria were performed. Of these, requests that were from the ORL department but for non- otorhinolaryngology diagnoses were excluded (*n* = 930)—for example, requests for head and neck alterations of traumatological origin and neurosurgical pathologies (tumours of the central nervous system, neurodegenerative diseases or cranioencephalic trauma). Requests carried out within the context of clinical trials and/or cancer treatments were also excluded (*n* = 439). Finally, patients whose clinical history could not be accessed (*n* = 24) were excluded. A total of 538 imaging test requests were finally included in the study.

The only forum is the Head and Neck Tumour Committee. It is made up of specialists in otorhinolaryngology, maxillofacial surgery, radiology, medical oncology, radiation oncology and anatomopathology. They meet weekly and they only discuss the imaging test referrals for tumoral pathology. None of the requests for other departments are discuss in the Committee.

### Data collection procedure

All imaging tests performed are included in the Radiological Information System (RIS/PACS) with a patient identification number. This database allows selecting all the tests performed in the Health System, according to patient, type of diagnostic test and diagnosis. Once the selected tests were identified, the medical records were obtained, and researchers used them to collect data retrospectively. Each clinical history associated with this test was reviewed to analysis the diagnosis and the justification included in the request. The rest of the information on the patient’s sociodemographic characteristics and imaging tests were also collected from the clinical history.

Patients were individualised by means of dissociated, non-identifiable codes that were nonsensical for any other information system and did not allow the identification of individual patients or their matching with other databases. The project database did not contain any data that allowed the identification of patients, and the research team did not have any patient identification information—whether from these databases or from other sources.

### Variables

The following explanatory variables were included: (a) Hospital; (b) patient’s demographic data (age, sex); (c) type of imaging test; (d) any other imaging test performed in the previous 12 months; (e) patient setting (hospital or primary care centre); (f) requested department (ORL, other clinical departments, surgical, emergency department, general partitioners and paediatrics); and (g) diagnostic suspicion (according to the Tenth Revision of the International Statistical Classification of Diseases- ICD-10-. The diagnostic suspicion was reclassified into eight groups according to anatomical criteria and pathology type for descriptive analysis and depending on whether the pathology was a tumour or not, for the multivariate study).

### Determining imaging test appropriateness

Imaging test appropriateness was determined by four expert researchers (specialists in ORL, anaesthesia, preventive medicine and public health) through a common protocol previously established. This protocol was developed based on the following guidelines: 1. Radiation Protection Guide 118, 2. Clinical Practice Guidelines of the American Academy of Otolaryngology Head and Neck Surgery, 3. American College of Radiologists Criteria Guidelines, and 4. ¨Do Not ¨ Guidelines of the Spanish Society of Radiology and Otolaryngology [9, 14, 18].

As a previous study did [11], the appropriateness of the tests was determined in accordance with the following criteria: (1) Appropriate: if the requested imaging test was recommended by the guideline; (2) Inappropriate: if the imaging test was not recommended for that specific clinical condition; (3) Not appropriately justified: if the requested form did not include sufficient information to establish the patient's clinical condition and diagnostic suspicion, therefore to enable assessing the appropriateness based on established recommendations; and (4) Not included in the guidelines: if the request did not correspond to any clinical scenario described in the guidelines.

To conduct the assessment, a pilot test was firstly performed. The four researchers independently evaluated 25 requests, and in a subsequent meeting, they discussed each request, assessed any discrepancies, and clarified any aspects in the initial protocol that required modifications. Once a consensus was reached and the new criteria to evaluate the rest of the requests were established, they continued with the independent evaluation of the requests. For each series of 100 evaluated requests, results were pooled, and possible discrepancies were assessed until a consensus was reached in accordance with the protocol. When doubts arose among the experts regarding appropriateness, the Radiation Protection Guide 118 was established as a reference, as well as three other resources if this latter Guide did not include recommendations for that specific clinical scenario. The protocol was thus updated. The associated radiation effective dose was estimated using evidence previously published [19].

### Data analysis

We calculated the prevalence of the four outcome categories and the associated effective radiation dose of the whole population, as well as the prevalence according to relevant variables. To compare each category with the selected patients' characteristics, the *χ*2 test or Fisher's exact test was used.

We estimated the relationship (OR and 95% CIs) between a synthetic binary outcome (appropriate vs non-appropriate, and this included inadequate according to some guidelines and not adequately justified) and the variables included in the study through an unconditional logistic regression. After evaluating possible interactions between variables and performing all possible two-way tests, the final multivariable model considered all the variables that were significant in univariate analyses (*p* < 0.05) and used a stepwise forward selection. All the analyses were carried out using the statistical software Stata V.11.

## Results

Table 1 shows the distribution of the variables according to test appropriateness. Of the 538 test requests, 42% were considered appropriate. The test requested by primary care were more likely to be considered inappropriate than those requested from the hospital (77.9% vs 19.1%, *p* < 0.001). Although tests requested by the ORL department were more likely to be considered appropriate (64.9%), 13% of requests were considered inappropriate. Tests requested by the general partitioners were more likely to be considered inappropriate compared to other departments (77.2%, *p* < 0.001).

**Table 1:** Description of the study variables according to the classification of the imaging test appropriateness

Variables	Total *n* = 538 (100%)	Appropriate 226 (42.0)	Inappropriate 185 (34.4)	Not appropriately justified 64 (11.9)	Not included guidelines 63 (11.7)	*p*-value
Hospital						0.004
Elche	250	122 (48.8)	69 (27.6)	26 (10.4)	33 (13.2)	
Orihuela	288	104 (36.1)	116 (40.3)	38 (13.2)	30 (10.4)	
Sex						0.283
Female	273	105 (38.5)	100 (36.6)	37 (13.6)	31 (11.4)	
Male	265	121 (45.7)	85 (32.1)	27 (10.2)	32 (12.1)	
Age (median, interquartile range)		55 (38–70)	44 (31–58)	60 (43–71)	52 (41–63)	< 0.001
Patient setting						< 0.001
Primary care centre	140	12 (8.6)	109 (77.9)	15 (10.7)	4 (2.9)	
Hospital	398	214 (53.8)	76 (19.1)	49 (12.3)	59 (14.8)	
Referrer department						< 0.001
Otolaryngology	185	120 (64.9)	24 (13.0)	10 (5.4)	31 (16.8)	
Clinical departments	68	20 (29.4)	21 (30.9)	16 (23.5)	11 (16.2)	
Surgical departments	14	6 (42.9)	3 (21.4)	3 (21.4)	2 (14.3)	
General practitioners	127	12 (9.4)	98 (77.2)	15 (11.8)	2 (1.6)	
Emergency department	124	66 (53.2)	28 (22.6)	17 (3.7)	13 (10.5)	
Paediatrics	12	1 (8.3)	9 (75.0)	0	2 (16.7)	
Diagnostic suspicion						< 0.001
Non-tumour head and neck pathology	40	7 (17.5)	7 (17.5)	10 (25.0)	16 (40.0)	
Tumour cervical	56	36 (64.3)	2 (3.6)	10 (17.9)	8 (14.3)	
Benign thyroid gland	37	12 (32.4)	21 (56.8)	1 (2.7)	3 (8.1)	
Tumoral thyroid gland	6	6 (100)	0	0	0	
Non-tumoral salivary gland	3	1 (33.3)	2 (66.7)	0	0	
Tumoral salivary gland	7	7 (100)	0	0	0	
Non-tumoral nasal pathology	295	97 (32.9)	147 (49.8)	23 (7.8)	28 (9.5)	
Tumoral nasal	26	20 (76.9)	2 (7.7)	1 (3.8)	3 (11.5)	
Infectious pathology of the ear	30	28 (93.3)	0	0	2 (6.7)	
Non-infectious pathology of the ear	20	12 (60.0)	4 (20.0)	1 (5.0)	3 (15.0)	
Type of imaging test						< 0.001
X-rays sinus	234	63 (26.9)	143 (60.7)	19 (8.1)	10 (4.3)	
CT Cervical	157	70 (44.6)	33 (21.0)	30 (19.1)	24 (15.3)	
CT Ears	51	40 (78.4)	3 (5.9)	2 (3.9)	6 (11.8)	
CT sinus	96	53 (55.2)	7 (7.3)	13 (3.5)	23 (24.0)	
Other radiology test performed in the previous 12 months						0.007
No	491	210 (42.8)	160 (32.6)	58 (11.8)	63 (12.8)	
Yes	47	16 (34.0)	25 (53.2)	6 (12.8)	0	

Requests in which the diagnostic suspicion was related to benign thyroid gland pathology (mainly goitre) (56.8%) and non-tumour nasal pathology (rhinitis, minor trauma) (49.8%) were more likely to be considered inappropriate. In addition, requests that included non-tumour head and neck pathology were more likely not to be included in the available recommendations (40%) (*p* < 0.001).

According to the type of imaging test, sinus X-rays were more likely to be considered inappropriate (60.7%) and ear CTs presented a greater probability of being considered appropriate (78.4%) (*p* < 0.001).

The multivariate analysis (Table 2) showed that the imaging tests requested from the Hospital of Orihuela were less likely to be classified appropriate (aOR: 0.40; 95% CI: 0.24–0.68) than those by the Hospital of Elche (*p* = 0.001). The same applied to requests by general partitioners (aOR: 0.18; 95% CI: 0.06–0.50, *p* = 0.001) and clinical departments (aOR: 0.27; 95% CI:0.11–0.60, *p* = 0.001) with respect to those by ORL department. When the diagnostic suspicion included a tumour pathology, imaging tests were more likely to be classified as appropriate (aOR: 7.12; 95% CI: 3.25–15.61) compared to the diagnostic suspicion of non-tumour pathology. The same applied to ear CTs (aOR: 17.16; 95% CI: 4.24–69.42, *p* < 0.001) and sinus CTs (aOR: 5.44; 95% CI: 2.10–14.13, *p* = 0.001) with respect to sinus X-rays. There were no differences according to age, sex, or having had a radiology test in the previous 12 months.

**Table 2:** Crude (cOR and 95% CI) and multivariable^a^ (aOR and 95% CI) association among imaging test appropiatness^b^ and the variables of the study

Variables	cOR	95% CI	*p*-value	aOR	95% CI	*p*-value
*Hospital*						
Elche	1.00			1.00		
Orihuela	0.52	(0.36- 0.76)	< 0.001	0.40	0.24–0.68	0.001
*Sex*						
Female	1.00		0.063			
Male	1.41	0.98–2.02				
Age	1.01	1.00–1.02	0.008	1.01	0.99–1.02	0.070
*Referrer department*						
Otolaryngology department	1.00			1.00		
Clinical departments	0.15	0.08–0.30	< 0.001	0.27	0.11–0.60	0.001
Surgical departments	0.28	0.08–0.93	0.038	0.54	0.11–2.61	0.441
General partitioners	0.03	0.01–0.06	< 0.001	0.18	0.06–0.50	0.001
Emergency department	0.41	0.24–0.71	0.001	0.71	0.41–1.13	0.058
Paediatrics	0.03	0.01–0.25	0.001	0.29	0.03–2.97	0.294
Diagnostic suspicion						
Non-tumoral	1.00		< 0.001	1.00		
Tumoral	6.32	3.49–11.47		7.12	3.25–15.61	< 0.001
Type of imaging test						
X-ray sinus	1.00			1.00		
CT cervical	2.84	1.81–4.44	< 0.001	1.76	0.67–4.60	0.250
CT ears	20.44	7.71–54.16	< 0.001	17.16	4.24–69.42	< 0.001
CT sinus	6.77	3.75–12.23	< 0.001	5.44	2.10–14.13	0.001
Other radiology test performed previous 12 months						
No	1.00					
Yes	0.53	0.28–1.01	0.053			

^a^Adjusted by hospital, age, referral service, diagnostic suspicion and type of imaging test

^b^Appropriateness was the reference category versus inappropriateness and not appropriately justified (imaging test for which the outcome was not included guidelines were excluded for this analysis

Table 3 presents the results of collective and per capita effective dose according to imaging test appropriateness. The collective cumulative effective dose for the 538 tests analysed amounted to 877.8 mSv. A total of 37.3% of the collective dose was associated with tests that were considered as inappropriate.

**Table 3:** Collective and *per capita* effective dose according to imaging test appropriateness

Variables	Total	Appropriate 226 (42.0%)	Inappropriate 185 (34.4%)	Not appropriately justified 64 (11.9%)	Not included guidelines 63 (11.7%)
Collective effective dose (mSv)	877.8	537 (61.1%)	327 (37.3%)	175.8 (20.0%)	165 (18.4%)
*Per capita* dose (mSv) (media, standard deviation)	2.3 (1.3)	2.4 (1.2)	1.8 (1.1)	2.7 (1.4)	2.6 (1.3)

## Discussion

Our results showed that only 42% of the imaging test requests were considered appropriate in accordance with the available guidelines. Differences were found between the two hospitals under study, the requested service, diagnostic suspicion, and type of imaging test. Almost 25% of the requests evaluated either failed to provide sufficient information or the diagnostic suspicion that motivated the request was not included in the available guidelines. In addition, over 30% of the collective dose corresponded to inappropriate imaging tests.

Vilar-Palop J et al. [11] analysed all the requests of two tertiary hospitals and their primary care centres in Spain regardless of clinical speciality. They found a frequency of inappropriate imaging tests similar to that obtained in the present study. They also encountered a similar frequency of tests that could not be classified due to lack of information by the requested clinician. However, previous studies have shown a wide range of frequency of inappropriate imaging tests when they focused on a particular disease or clinical department. A systematic review focusing on cardiovascular pathology showed that between 7 and 23% of transthoracic echocardiograms performed were considered inappropriate; between 28 and 30% of stress echocardiograms were classified as inappropriate, together with 9% to 44% of cardiac CT studies and 4% to 46% of SPECT studies [20]. Moreover, a study which evaluated the appropriateness of imaging test requests in an emergency department showed that only 15.3% of the requests contained sufficient clinical information, a slightly higher percentage than that found in our own study [21].

In contrast with the present study, no differences had previously been found between hospitals of a similar category [11] or between hospitals of a different category [22]. In our study, both hospitals had the same radiological resources, and the only difference between both hospitals was that the hospital that showed a greater frequency of appropriate imaging tests was a university hospital.

We also found high percentages of inappropriateness in the requests that included simple X-rays, mainly of paranasal sinuses. Similarly, other studies have also shown a high percentage of inappropriateness of requests for simple X-rays in the domains of the abdomen, lung, and lumbar spine [9, 11]. This high frequency of inappropriateness could have several causes. Firstly, the lack of clinicians’ awareness of the available guidelines as has been shown previously [11]. In this sense, interventions directed towards raising clinicians’ awareness of radiological guidelines has led to an increase in the percentage of requests considered appropriate [22, 23]. Requests by general practitioners showed the highest frequency of inappropriateness and the short time in their practice for attending each patient could also be a potential cause. Finally, there are stricter requirements for ordering a CT than for an X-ray test, which could explain the high frequency of inappropriate X-rays.

Many of the imaging tests studied failed to include sufficient clinical information in their requests and hence, we could not classify them. To include clinical information in the request is critical for radiologists when interpreting an imaging test and according to the European directives, it is also a legal requirement. Thus, different strategies should be developed to implement the justification of an imaging test. In this sense, to improve clinicians’ knowledge regarding imaging tests or to implement clinical guidelines and audits, have previously generated positive results [22, 23]. In the same way, although the existing recommendations include the most frequent diagnostic suspicions in clinical practice, it is necessary to continue developing recommendations that cover as many clinical situations as possible. This would help to perform appropriate imaging tests and to reduce population’s exposure to the associated radiation, which is significant.

The study has several limitations. First, the retrospective nature of the medical records must be considered when interpreting the results. We based our classification on the available data and may have missed some data in the medical histories that could have contributed to less accurate classifications. Secondly, we used the available guidelines as a reference to assess imaging test request appropriateness. This approach was simple but presented limitations because it did not allow us to evaluate each individual patient’s situation. Therefore, our results may underestimate the percentage of appropriateness. Nevertheless, each imaging test was independently reviewed by four researchers who were experienced in this type of evaluation and when a disagreement arose, a single consensus solution was reached. Head and neck cancers involve a variety of symptoms which can also be found in non-malignant disorders [24]. The wide range of symptoms suggest that the clinical presentation of these cancers is very unspecific, thus hindering the diagnosis of these cancers. Previous data showed that cancer was suspected by otolaryngologists only in a relatively small proportion of patients and approximately 23% of individuals received a diagnosis of suspected cancer during the year prior to the confirmed pathology [25]. Thus, in our study, the prevalence of tumoral diagnostic suspicion may have been less than that which occurs in clinical practice. According to previous evidence [26], in our study the initial diagnostic suspicion was based on an irregularly infiltrating mass with or without ulceration. In addition, the probability of having received a diagnosis of suspected cancer was associated with several demographic and clinical variables, such as age, sex and the presence of dysphagia, dysphonia and other symptoms, as previously was shown [25]. Lastly, both hospitals were planning to replace sinus X-rays by low-dose CT of sinus in 2022, for both ORL and maxillofacial services. However, this was delayed due to the COVID-19 pandemic.

Our study provided an overview of the use of imaging tests associated with radiation in ORL and showed that the frequency of inappropriateness was associated with the requested department (general partitioners and other clinical departments), diagnostic suspicion of a non-tumoral pathology and sinus X-rays. In addition, despite the directives issued by Europe and the efforts made from several settings to increase the justification for an imaging test, there is still a high percentage of tests that do not include sufficient clinical information.

## Data Availability

All data relevant to the study are included in the article.

## Abbreviations

CT: Computerized tomography
FDA: Food and drug administration
ORL: Otolaryngology
